# “They want you to know who they really are inside of the old visage”—biographical storytelling as a methodological tool to explore emotional challenges in old age

**DOI:** 10.1186/s12877-023-04094-8

**Published:** 2023-06-23

**Authors:** Chao Fang, Alastair Comery, Sam Carr

**Affiliations:** 1 Department of Social and Policy Science and Centre for Death and Society, University of Bath, 3 East, Bath, BA2 7AY UK; 2 Department of Education and Centre for Death and Society, University of Bath, Bath, UK

**Keywords:** Biography, Storytelling, Older people, Ageing, Emotional challenges, Qualitative methods

## Abstract

**Background:**

Growing older is often associated with resilience, contentedness and inner growth. Older people however are also at risk of confronting unique emotional challenges as a result of varied ageing-related experiences. By employing a biographical lens, we aim to introduce storytelling as a methodological tool to more holistically explore older people’s emotional challenges and to improve their wellbeing.

**Methods:**

Building upon theoretical understandings about the narrative construction of identity across the life span, we draw upon a qualitative study about older people’s loneliness as an example to showcase the methodological value and feasibility of biographical storytelling. We aim to better understand the nuanced and sometimes painful emotional experiences that can be encountered alongside ageing.

**Results:**

Findings from the qualitative study we showcase, highlight that unique emotional pains and the (in)ability to deal with such in old age could be deeply rooted in older people’s earlier lives. These findings contextualise people’s emotional challenges and needs within their identity, as a narrative thread that links their past, present and expected future. As such, our example study shows that emotional challenges in old age are not only ageing-related, but can be more fundamentally connected to disruptions to the ongoing flow of narrative identity construction.

**Conclusion:**

The highly retrospective and reflexive nature of these findings illustrates the methodological merit of biographical storytelling. We argue that the impact of biographical storytelling can go further than both conventional semi-structured narrative interviews and existing interventional tools. Instead, it is a particularly useful research methodology to explore human experiences and needs in the unique context of ageing. This methodological development thus provides an insightful analytical lens to explore how older people’s earlier life experiences may be carried forward and confronted to shape their emotional stability in the present and future stages of their ageing lives. Beyond the methodological significance, we further demonstrate the benefits of empowering older people to reconstruct their ageing lives in the context of their biography.

**Supplementary Information:**

The online version contains supplementary material available at 10.1186/s12877-023-04094-8.

## Introduction

Growing older is often associated with resilience, contentedness and inner growth [1, 2]. On the other hand, older people may also embrace unique risk of confronting negative emotional experiences such as loneliness, sadness and anxiety [3]. Research has suggested that changes and challenges alongside ageing (e.g., bodily deterioration, loss of others and approaching mortality) can threaten to undermine older people’s emotional stability to retain resilience in their ongoing lives [4, 5]. These emotional challenges may further disrupt older people’s wellbeing by which they have long ensured the continuity and coherence of their identity as a meaningful entity [6, 7]. The complexity of these disruptions to the emotional lives of older people can be further compounded by broader social environments of cultural values, public attitudes to ageing and wider social and health support [2, 3].

Qualitative research methods have long paid attention to the complex aspects of the ageing process and older people’s lived experiences. The current methodological literature has largely focused on ‘involving’ and ‘empowering’ older people in research design, ranging from traditional interviews and focus groups to more participatory and creative methods, such as photovoice [8–10]. Storytelling has emerged as a scientifically insightful and emotionally meaningful tool that empowers older people to draw upon socially accessible discourses (e.g., memories, language, multimedia materials) to vividly reconstruct their lived lives for the researchers, and to further make sense of their experiences and promote wellbeing [11–13]. As highlighted by Brotman, Ferrer and Koehn [14], talking about life histories can “capture the complex and intersecting identities of older adults and expose the often-invisible institutional structures and relationships of power that mark older adults’ interactions with family, community, and the state in their everyday lives” (p.480).

The emotional challenges facing older people are not fragmented experiences unique to the ageing process, but rather, are often rooted in their lived lives. Therefore, it is of particular benefit to understand older people’s emotional challenges in the context of their biography. Incorporating such a biographical approach to understand older people’s challenges and responses in their emotional lives can afford researchers a retrospective and reflexive lens to explore older people’s “historically situated subjectivity” [11, 15]. That is, biographical storytelling in this vein is a suitable tool to collect and interpret how older people’s experiences of emotional challenges, as rich and complex composites, are interwoven by both their present ageing and their individual life histories, personal meanings and broader socio-historical contexts [16, 17]. This is also an empowering approach, through reflections on both ‘big’ (significant/life-changing) and ‘small’ (everyday) stories across the lifespan, older people can express both conscious experiences, and meanings and values that are deeply embedded in their ongoing lives [15]. Therefore, biographical storytelling has the potential to go further than simply being perceived as conventional semi-structured narrative interviews (focusing on one’s recent lived experiences) and interventional tools (seeking to therapeutically promote wellbeing). Instead, it can be a particularly useful research methodology to explore human experiences and needs in the unique context of ageing.

In this article, we draw on a large qualitative project about the loneliness of 80 older people in the UK and Australia, to demonstrate the methodological value and feasibility of implementing biographical storytelling in gerontology. We believe that this study is an insightful example of biographical storytelling. We view loneliness broadly as a condition that intersects with varied emotional challenges, including not only a sense of lacking meaningful relationships but also a fundamental sense of emptiness and meaninglessness [3, 18]. Our goal is to better understand the nuanced and sometimes painful emotional experiences faced by individuals as they age. We explore the following questions: 1) how biographical storytelling can enhance our understandings about people’s emotional challenges in the unique context of ageing, 2) how researchers can collect biographical interview data to provide a historical context for older people’s emotional challenges, 3) how we can interpret rich and retrospective narratives to comprehend their current emotional challenges and future concerns. Through our article, we aim to situate these experiences within their biographical contexts, considering the perspectives and meanings that older people attribute to them. This biographical storytelling method can also uniquely allow researchers to examine the implications of these experiences in their ongoing later life.

## Background

### Understanding biographical storytelling as a methodology in the context of ageing

Research has long argued for storytelling as an authentic methodological tool to capture enriched life encounters as experienced by individuals and intersected with wider socio-historical contexts [19, 20]. The value of storytelling in research lies not only in generating rich, in-depth data in the form of stories to inform research outputs; but it is also closely connected to and reinforced by the storytelling process per se that enables both the storyteller and the researcher to co-construct, compare and contrast their varied interpretations of how experiences are created and given meaning [21]. In a classic debate on the power of language, sociologist C. Wright Mills [22] asserted that language provides speakers with a means of accessing available linguistic and cultural discourses to ‘tell’ (more accurately, interpret and justify) their experiences, and how they negotiate these within their unique situational contexts (e.g., with listeners, in socio-historical environments). The power of storytelling can particularly be evident in a biographical context, in which the retrospective lens and its reflexive nature can afford people the opportunity to revisit their lived experiences from across their lifespan to further reconstruct their self-narratives over time. A range of methods and models, such as “life story Interview” [17, 23], “life-story work” [13] and “autobiographical memories” [24], have been developed thus far to highlight the benefits of reconstructing and restoring the coherence of self-narratives in ongoing lives. Key to these biographical methodological approaches is the focus on identity as a ‘story’ or more accurately a ‘plot’ that “a person can understand themselves as living through time, a human subject with a past, present and future, made whole by the coherence of the narrative plot with a beginning, a middle, and an end” (p.120) [25].

**Fig. 1:**
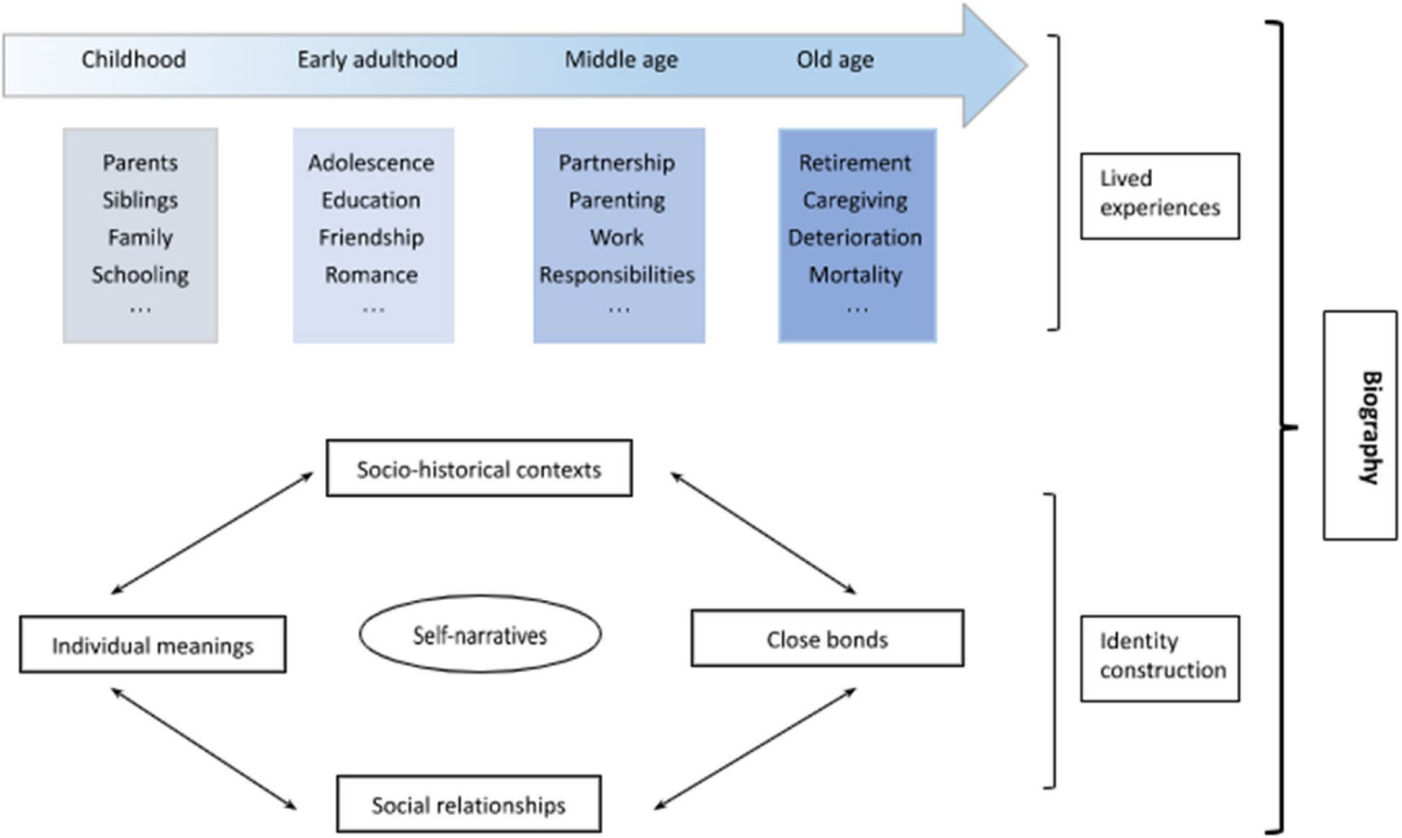
Theoretical construction of human biography

The narrative construction of identity lies in the theoretical foundation of biographical storytelling. As shown in Fig. 1, lifespan consists of varied phases, starting from childhood all the way to old age and ultimately terminating at the end of life [25]. Throughout their lifetime, humans experience and often learn from others about what is considered to be (or not to be) important, valuable and real at different stages [26]. Meanwhile, they also tend to internalise these experiences and knowledge (despite being positive or negative, for example, personal growth or trauma) to construct a thread of narratives to nurture and maintain their sense of self as a unique being [27]. These narratives are not always distinct but are often closely connected with each other, contributing to a sense of self-identity which can be understood and made meaningful over time [25]. As such, people’s past experiences can be habitualised as a complex of meanings and resources providing references for them to make sense of who they are at present and further envisage their future self [17]. As life moves on, this spatial–temporal construction of identity is also ongoing across the life stages, in which individuals encounter varied experiences and socio-cultural scripts to continuously affirm, revise and justify their self-narratives [26].

This understanding of identity as a narratively constructed and biographically interconnected entity can afford unique insights into the lived experiences of older people. This methodological value has been long discussed in narrative gerontology, by which life is seen as a story and the narrative construction of identity remains ongoing and active across the process of ageing [25]. As such, ageing presents unique opportunities to reflect on and appreciate their lived experiences to better make sense of and encompass their self-narratives. While acknowledging the positive impact of ageing, narrative gerontology also implies that biographical storytelling has particular relevance to emotional challenges in old age. As aforementioned, growing older may uniquely endure the risk of suffering from varied emotional challenges due to the multifaceted changes and losses alongside ageing [3, 14]. Narratively speaking, many of these emotional challenges may not solely result from ageing but also have historical roots in older people’s biography. For example, loneliness may be experienced as being separated from the world that older people had long taken-for-granted [3], while frustration may be connected to painful realisation of no longer being who they used to be [5]. As such, emotional challenges in old age may arise from ruptures that disrupt the ongoing construction of self-narratives as a coherent entity across the lifespan. Similarly, older people’s ability and resilience to cope with these challenges may also be significantly informed by their life histories. To better understand and support older people’s emotional challenges, therefore, it is useful to link their ageing lives to and within their biography as a system of narratives imbued with individual life histories and intersected with broader socio-historical backgrounds [13].

Existing biographical methods/models can certainly provide a valuable direction to develop biographical storytelling as an authentic methodological tool to explore older people’s emotional challenges. A good example is the ‘life story interview’ method, which provides a thorough structure to allow for sensitive collection of a (fairly) compete narrative of one’s lived experience, highlighting important life events [23]. Meanwhile, the ‘life-story work’ [13]and ‘autobiographical memories’ [24]models have paid more direct attention to reviewing life in the context of ageing. They develop both methodological and interventional tools to explore how reflexively recollecting, evaluating and ascribing meaning to memories can help to improve cognitive ability, mental health and wellbeing (particularly in old age and during times of changes). Whilst the above methods/models have shown how in-depth life review interviews can capture the ongoing construction of self-narratives and how the sense of self-continuity can inform future identity development, a dedicated focus on the unique and often sensitive nature of emotional challenges in old age is required. We believe that ageing is closely connected to greater life experience and development (e.g., problem-solving competence, calmness and a different view on life). Due to the natural process of ageing, older people may also face the prospect of physical decline, loss of others and even their own approaching mortality, which are likely to give rise to varied emotional challenges. Existing models have either adopted a generalised approach to interview participants from varied age groups (without being tailored for unique life stages facing older people) [23]or have used story-telling as a pathway to intervention/treatment for older people [13, 24]. Therefore, it is key to note that the experience of telling one’s lived life is not only an empowering interventional experience, but also an accessible tool more inherently embedded as part of everyday life through which people can naturally express the authenticity of self alongside ageing. These experiences can subsequently enable these older people to reflect on their rich experiences, long accumulated across their life histories. These reflections can further illustrate the (dis)continuity of self-narratives, as a meaningful whole, to better understand their emotional challenges and ability/needs to address these within their biography.

To better understand older people’s unique emotional challenges, it is important to develop a tailored biographical storytelling method that focuses on their ageing lives within their life stories. We argue that biographical storytelling can provide insights into their habits and values, and how these may change and be lost as they age. By enabling older people to reflect on and express their experiences, emotions and their long taken-for-granted meaning within the interview context, this methodological approach can help researchers to develop comprehensive knowledge about participants’ (older people) life histories. Biographical storytelling can also offer older people a unique social context where they can sequentially and consequentially reconstruct and share their lived experiences (shaped by their long-held meanings and identities) [28]. While retelling life stories may have subjective constraints, it can provide an integrative tool to gain insight into older people’s experiences within their biography rather than as isolated events. Additionally, when exploring emotional challenges, careful attention should be given to framing and organising interview questions to ensure a safe and insightful exploration of older people’s lived experiences. In the following sections, we use the Bath Loneliness Study to demonstrate how a biographical storytelling approach can sensitively capture and centre older people’s emotional challenges within their life stories.

## Methods

The example we draw upon here is a large qualitative study entitled “the Bath Loneliness Project”. The project was conduct between 2019–2021, to use loneliness as a lens to explore the emotional challenges facing older people living in the UK and Australia (for further details about the project findings, please check [3, 5]). These two countries were chosen as both have seen significant socio-demographic shifts in recent years, with increasing numbers of older adults living longer and healthier lives. In addition, increasing emphasis on healthy ageing and life-long development in both countries also provided an ideal context to explore the ongoing narrative construction of older people’s biography and how their emotional challenges may fit into the process [29]. The understanding of loneliness we adopted in this study was not just simply feelings associated with lacking meaningful relationships and interactions [18]. Instead, we understood loneliness more broadly as a condition deeply imbued with older people’s emotional lives in which their feelings and moods are intersubjectively shaped by changes both in individual experiences and socio-cultural dynamics over the course of their lifetime [30]. As such, this broadly defined loneliness, as demonstrated in our other work [3, 5], has afforded us an insightful perspective to explore how varied emotional challenges can be prompted, shaped and supported by older people’s biographical artifacts and how loneliness can encapsulate emotional challenges within their ongoing construction of narrative identity [25]. The insightfulness of loneliness as a concept encompassing emotional challenges in old age is also highlighted by the cross-cultural comparison, in which we found emotional challenges are risks facing older people despite their social situations (although we are not suggesting there are no differences between the UK and Australia, given the objective of this methodological article, we seek to explore the value of biographical storytelling in a wider context).

### Interview design

Biographical storytelling was employed and operationalised in the form of in-depth and semi-structured interviews. This design is distinctive from conventional research interviews, which often focus on participants’ experience at present or in recent time. Instead, we designed our interview with older people to retrospectively explore their emotional lives across the lifespan and how these could shape their emotional challenges in old age. As such, semi-structured interviews were used as a socially constructed vehicle encompassing and organising the dynamic aspects of how these older people engaged with scripts at different levels and phases of their lives to deal with and make sense of their emotional voids and losses [31]. Adopting a biographical lens, we encouraged older people to lead the conversation to tell their stories containing the following aspects (not necessarily in a strictly chronological order): (1) their lives up to this point (including childhood, adolescence, career, family, and anything else they wished to share and discuss), (2) their closest relationships, (3) their experiences of loss, (4) feelings of loneliness and other associated emotional challenges, (5) their current living environments, and (6) their future concerns/plans. Such a structure is by no means limited to these topics and a biographical approach of this nature would encompass a wide breadth of research interests.

The interview questions focused on each of the above areas, such broad outlooks enabled an enriched interview, where the intertwined nature of topics often became evident and sections often intersected with each other (see Additional file 1: Appendix 1). A narrative approach was adopted, empowering the older people to freely tell their stories around these core subjects across their entire lifespan [32]. These questions were used as guides rather than verbatim, providing the interviewer with a thread of topics and directions to assist the interviewee (older people) to verbally reconstruct their ongoing emotional lives. We were not intended to capture their emotional lives in ongoing lives as a linear process. Instead, we deliberately designed the interview questions to contextualise and focus on interviewees’ lived experiences of their emotional lives and challenges in old age within the ongoing development of their identity as narratively constructed and biographically interconnected entity. In so doing, we also aimed to minimise the distress of the storytelling experience and to more holistically understand their emotional challenges and needs in old age.

### Interview collection

In light of the carefully designed interview schedule as outlined in Additional file 1: Appendix 1, all the interviewers were professionally trained in sessions organised by the Principal Investigator of the study (the third author) prior to the interviews. Five researchers, including the three authors, conducted semi-structured interviews with 40 participants in the UK and the other four researchers interviewed the remaining 40 participants in Australia (see Table 1). Approximately 8,000 min of rich audio data with the 80 older people were collected from both countries between 2019–2020. These Interviews ranged from 70 to 200 min and averaged around 100 min, capturing an in-depth picture about how older people’s emotional challenges and subsequent responses could be deeply rooted in their earlier life experiences. The interviews also highlighted the responsiveness of the interviewees to such a biographical method. As clearly evidenced, these older people were often very receptive to the lines of questioning, resulting in rich biographically informed data.

**Table 1:** Socio-demographical characteristics of interview samples

Characteristics	Sample (*N* = 80)
Age (years)	
55–60	2
61–70	9
71–80	35
81–90	31
91 +	3
Gender	
Female	55
Male	25
Marital status	
Divorced/separated	7
Married/partnered	26
Unmarried	4
Widowed/widowered	43
Health	
Has a chronic condition(s)	28
Healthy	52
Household	
With the spouse/partner	41
Living alone	37
With friends	2

### Reflexivity

This biographical storytelling method allowed us to create a collective transformational space, to not only co-construct knowledge about emotional challenges in old age but also to deepen understandings about the value of reviewing lived experiences in life histories [33]. It was only possible for us to collect such rich biographical interview data because of a trusting relationship between us and the participants. This trusting relationship was often developed in the conversations we had about their experiences of relationships, challenges and support at different life stages. Such conversations fostered a sense of empathy, by which we could more closely understand the older people’s feelings based on experiences of ourselves and our close others (e.g., our own (grand)parents). The experience of conducting the biographical storytelling interviews afforded us as researcher a transformational experience, granting us an insightful and vivid lens (not only for data analysis but also in our everyday lives more generally) to appreciate the value of both understanding the life stories of others and sharing our own. Despite the above benefits of being embedded in the biographical storytelling process with the older people, we were also aware of the potential risks of being overwhelmed by the relationships and the data we developed from the research. Therefore, regular meetings were held between the team members to debrief and resources on further/professional support were also provided throughout the project.

### Data analysis

We adopted a reflexive thematic analysis method to identify, analyse and interpret patterns of meaning about these older people’s emotional challenges within their biography [34]. An inductive approach was embedded throughout to allow us (as researchers and also as ‘strangers’) to be involved in a reflective and iterative process of data analysis. This enabled us to reflect on our own subjectivity and actively engage with these reflections throughout the analysis to capture the older people’s lived experiences and nuanced feelings from their own perspectives, without being dominated by the assumptions and our pre-existing frameworks (however, we also recognise that thematic analysis is an inherently subjective process that may involve potential bias and theoretical preferences). We followed the six-phase thematic analysis process recommended by Braun and Clarke, which involved familiarizing ourselves with the data, generating initial codes, searching for themes, reviewing and discussing potential themes, refining and naming themes, and writing themes up. The analysis was conducted by all authors, who read the interview transcripts and notes thoroughly before conducting independent coding of each interview transcript. A combination of NVivo 12, a qualitative analysis software package, and more traditional reading and coding was used to manage and analyse the data. The authors met frequently to discuss and compare findings and codes. If there was disagreement or divergence on codes, further reading and discussion were conducted until a consensus was met.

**Fig. 2:**
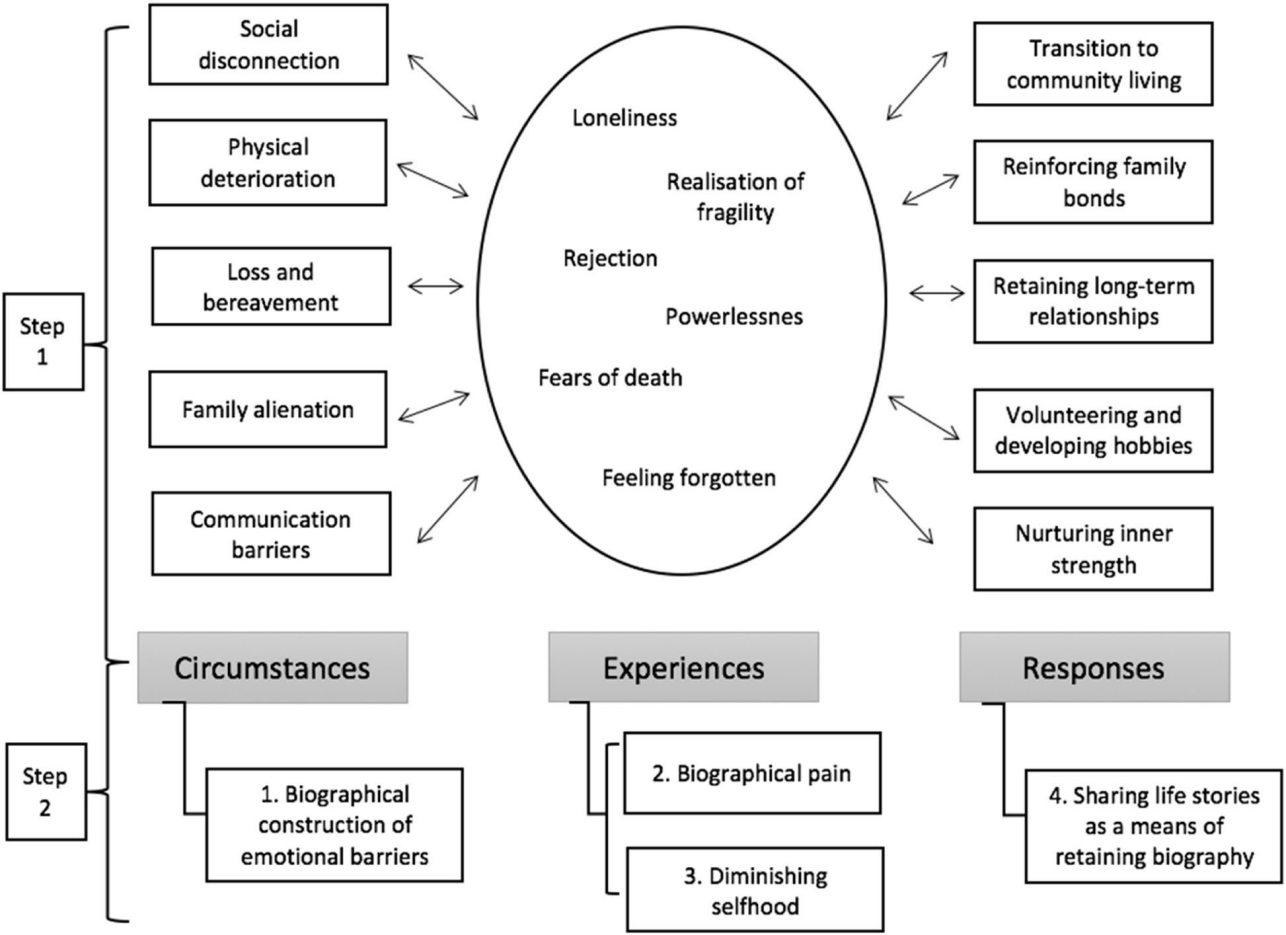
An illustration of the coding process

To more systematically capture the complex experiences of emotional challenges in old age, we adopted a generalised organising framework from Ettema et al. [18], to contextualise our findings in three dimensions: (1) *circumstances* from which emotional challenges may arise, (2) *experiences* of multifaceted emotional challenges (3) *responses* to deal with these challenges alongside ageing. This categorisation also mirrors our social constructionist ontological standpoint and interpretivist epistemological approach, allowing us to engage with our interviewees’ accounts to capture how their rich lived experiences of emotional challenges in old age are interpreted and constantly constructed by the ongoing development of their biography. The thematic analysis lens enabled us to identify and systematically summarise our rich findings about older people’s emotional challenges in the complex context of their life histories and broader society. Figure 2 shows our coding process. Step 1 captures an illustration of an early iteration of coding, in which older people’s reported experiences, both in old age and earlier life, were analysed against the research questions (on biographical storytelling as tool to more holistically understand older people’s emotional challenges) and were further categorised under the three dimensions mentioned above. Step 2 was conducted for this article, aiming to re-categorise a wealth of qualitative findings to methodologically showcase how a biographical lens can be applied to more comprehensively analyse older people’s emotional challenges. By employing our study as an example, we seek to illustrate why it is both important and useful to interpret older people’s life stories not as a series of segmented experiences but as rich and complex composites “embedded in a coherent, meaningful context, a biographical construct” [35] (p.3).

## Results

We contextualised the older people’s varied emotional challenges (often broadly manifested as loneliness or other associated conditions such as emptiness and/or meaninglessness) in the ongoing process of their biography as a narratively constructed entity. We found, the boundaries between past, present and even future were fluid and interconnected [36]. As such, we sought to explore how both the explicit and hidden meanings of their experiences, not as a completely new composite but rooted in their biography, have been continuously constructed and negotiated alongside their life course. Four interconnected themes have been developed for this section: (1) biographical construction of emotional barriers, (2) biographical pain, (3) diminishing selfhood and (4) sharing life stories as a means of retaining biography. Theme 1 underlines the biographical circumstances that gave rise to emotional challenges in old age. Theme 2 and 3 capture the experiences of how life histories may persist, overshadowing older people’s emotional lives and conversely how losing these lived lives alongside ageing could also prompt deeply painful emotional distress. Theme 4 highlights the older people’s responses to various emotional challenges and how their emotional strength and growth were fostered or reaffirmed during the interview process. In so doing, we aimed to show the significance of retaining the integrity and consistency of biographies and how older people sought to do so by interacting with socio-cultural scripts and by constructing individual meanings. To protect the confidentiality of our participants, pseudonyms are used throughout and all identifying materials are removed.

### Biographical construction of emotional barriers

Lives are constructed and made coherent by narratives that provide a plot of stories to integrate one’s past, present and anticipated future into a meaningful whole [25]. Our analysis revealed that emotional challenges, such as loneliness and other associated emotional challenges, may arise from an array of barriers that create disruptions to the coherency of older people’s biography in their ageing lives. These barriers were often inherently emotional and deeply intertwined within their life histories. That is, changes and losses in these older people’s present lives could prevent them from drawing upon once taken-for-granted resources to meaningfully express, communicate and further manage their emotions.

One of the most prominent emotional barriers reported in our participants’ accounts was the challenging circumstances of losing people and things that have long helped them retain meanings and resources central to their identity. More than half of our participants had recently lost their spouse and other close families/friends. By analysing their reported experiences connected to their life histories, we found that these losses might not only prompt emotional voids of attachment, security and connectedness in old age, but could also shatter the long-term emotional resources that they had always employed to (re)affirm their taken-for-granted lives and identities. Paula (aged 72) felt she was nobody after losing her husband whom she had long lived with and cared for:

Paula: “*[W]hile he was alive and I was his full-time carer, companion, friend, we had a ball even though he was in a wheelchair, but when he was gone I didn’t know where I fitted anymore. I didn’t know who I was anymore because I wasn’t…”*

These emotional barriers could also arise from the loss of good health and physical capabilities that to date had determined their strength in fulfilling their roles and responsibilities in their lived lives. After suffering a stroke, David (aged 80) strongly conveyed his emotional vulnerability due to the changing caregiving roles between him and his wife and ultimately about the loss of his ‘old’ self as a healthy and capable husband:

David*: “Well, my wife has always had a difficult hip and right from the days of ironing her blessed skirts I’ve been the one who can do things. I just got on with it… But now, it’s her who has to put my shirt on me. And at this present time, with my left arm sitting pretty well useless in my lap, I’m finding it so, so difficult.”*

The risk of losing one’s emotional strength was not only attributed to losing biographical resources to retain emotional strength but might also be a more direct result of experiences from earlier life. By embedding their varied emotional barriers in the ongoing process of their lives, we discovered that many older people (especially men) embodied a ‘stiff upper lip’ mentality, which reflects unique cultural, generational, gendered and familial circumstances many of the older people shared in their earlier life. Like many others, Simon (aged 92) showed us how older people’s abilities to express emotions and seek support could be historically smothered and how this could undermine their strength in facing varied challenges in old age:

Simon: “*You had to keep a stiff upper lip as it was called and I suppose that’s stuck with me. I mean, people in my generations who came through the war, not that we were fighting, I was too young, a child, a boy, you had to get on with all sorts of things… [but] I feel sorry for myself at times when you are sat and you think oh god, I do wish she [my wife] was here and I do miss her.”*

From a methodological perspective, we were afforded an insightful and retrospective lens to illustrate how the barriers (circumstances) facing our participants when dealing with their emotions in old age might intersect with their prior lived experiences. This analytic approach allowed us to more fully elucidate the significance of our findings not just in the now but across the life span of the older people, making succinct connections between events across their biography in relation to their current emotional challenges. In so doing, we were then provided a platform, from which to explore the older people’s experiences and responses to these varied emotional challenges in the ongoing construction of their biography moving forward (as shown below).

### Biographical pain

By conducting an in-depth analysis of these older people’s life stories, we were able to capture some profound emotional pains that they carried with them from across their life course and that could become increasingly difficult to ‘put right’ (as older people lose close others and things key to their emotional strength alongside ageing) [37]. Whilst sharing the benefits and contentedness of growing older, these older people often also hinted at the (re)surfacing of painful and distressing narratives and memories that had previously been buried in the busyness of independent living and excitement of being ‘young’.

When reflecting on their lived lives, the older people often reported traumas and regrets in the past deeply ‘engraved’ on their biography. Such deep pains might remain or even intensify in old age, as reported by Jane (aged 80) who spoke about her the insecurity and lack of confidence due to mistreatment during childhood that continued to haunt her emotional life in old age:

Jane: “*So from an early childhood I learnt that I was a bad person. My brother certainly told me that. I was stupid, ugly, etc… I mean, last year my daughter-in-law, the French one, started suddenly explaining to me what a worthless person I was and I burst out in tears and it took me months to think, well, poor you, if you are not able to like me, how horrible must that be for you, how horrible to hate.”*

As a result of losing resources to retain emotional strength in old age, the regrets accumulated in earlier life could be revived, deeply questioning the worthiness of their lived lives and the integrity of self. Patricia (aged 78) reflected on ‘being a loner’, which had long been considered by her as a strength but now became a significant emotional pain as she grows older.

*Patricia: “I look back and think I was a loner throughout my life, but that’s the way I wanted to be. And if you didn’t accept me as that, or didn’t include me like that, I’ve learnt in the past, ‘Well, I don’t care anymore,’ but now I do care when children call me ’single’ and ‘old’. But yes, I’ve been rejected by children, which is the worst thing; it’s horrible.”*

Such biographical pain was vividly illuminated by the storytelling approach outlined above. Key in our analysis was the focus on the coherence of human biography and how pain in earlier life may remain influential in old age and even how strength may gradually turn to deeply painful emotional challenges. By contextualising confrontation of profoundly deep pains in old age within the life course, we were afforded a biographical lens to more holistically and historically understand the deeper roots of the older people’s emotional challenges.

### Diminishing selfhood

Prior to this point we have elucidated how our method was used to extrapolate links to participants lived lives and to understand their emotional challenges in relation to their previous biographical experiences. This theme will now illustrate how the biographical construction of their current ‘self’ in relation to their past is carried forward and confronted in the future as they age. Integral to their emotional challenges was a realisation that their self-narratives were (sometimes increasingly) disrupted and had been irretrievably lost alongside their ageing [30].

After living a long life full of experiences and memories, many older people talked about their fear of fading away in a sense that their lives would be forgotten as they grow older. As experienced by Iris (aged 90), living to be old could prompt deep and often growing pains of feeling disconnected and alienated from the external world due to varied ageing-related losses:

Iris: “*The only other thing is of course that most of my friends are dead. I’m 90 on Christmas day and when I go through my life, my school friends, most of them are gone, my college friends, most of them are gone. I’m the only in-law left, I’m the only great grandparent left. So that aspect of the extended family becomes less and less… Yes, then it becomes lonely as regard your future and your past life and the people that I was familiar with, people that I worked with, of course, and the people that I had social contact with and relatives. My own family, I’ve only got one brother left and my husband was Dutch, and the Dutch family, one sister-in-law. So I’m finding that I’m standing alone as regards my former life.*”

The older people could further confront the emotional challenge associated with feelings that their lives no longer mattered and their biography was becoming increasingly unimportant. Emma (aged 77) highlighted her emotional struggles whilst trying to retain the authenticity of her own being:

Emma: “*Absolutely, and ‘who am I?’ This is why old people talk about their lives, their past, or whatever, because they want you to know who they really are inside of the old visage*.”

Underneath these emotional challenges of fading away, we found that there was also a more existential awareness of the approaching mortality and the finite nature of their lives. As they age and continuously confront a multitude of losses and challenges, many older people felt death closer than it had ever been before. Robert (aged 72) painfully realised that death was no longer a distant reality after losing his friends:

Robert: “*I did sort of think, a couple of years back, ‘Oh, I’m now at the age that Jim died.’ But it’s one of the things that you come to learn, these things happen in life, I’m afraid. There was a young lady, younger than us, again died of cancer. That was one of our bridge group. We sort of knew it was going to happen, but it happened quicker than was expected, and again, that brings home your mortality*.”

The rich lives lived by the older people, as illustrated above, could come under threat as a result of ageing, losing others and ultimately confronting the inevitable fate of death. Our analysis ascribed a deeper meaning to older people’s emotional challenges of loneliness, helplessness, worries and even fears. That is, we drew upon their biographical life stories to capture how their cherished memories and relationships (which have long defined who they are) could be left behind, thus causing deeply painful emotional challenges at a more existential level.

### Sharing life stories as a means of retaining biography

Employing our methodological approach of integrating older people’s narratives and experiences into their ongoing construction of biography afforded us a meaningful context to better understand why (circumstances) and how (experiences) these older people encountered varied emotional challenges alongside ageing. Such an approach also illuminated their dynamic responses to adopt, reject and revise existing resources to restore the continuity of their self-narratives over time, as a means of (re)negotiating in the face of various emotional challenges.

The above analysis has shown that the older people’s emotional challenges could be experienced in a pervasive and often deeply painful manner as a result of multifaceted losses threatening their taken-for-granted life routines. We found that the older people’s efforts to alleviate emotional challenges often focused on maintaining/restoring key resources (e.g., family connection, social and community support) that have long help them stay strong and consistent. The unique biographical lens we employed further illuminated a deeper dimension of their responses, that was to preserve the meaningfulness of their lived lives and their being, potentially in the face of painful memories and fading existence in the world. For example, after moving closer to his daughter and her family, Craig (aged 72) and his wife confronted a profound need to carry on their memories and identities in the family beyond the finitude of their lives:

*Craig: “We are creating a memory box for each of them [grandsons] where we’ve written a kind of autobiography each of our life, the interesting bits anyway, the bits that we want them to know… We are finding it quite cathartic and quite nice and I would like to think that in 50 years’ time, our grandson will show this information to his grandchildren.”*

Some older people also demonstrated a concern with passing on their lived experiences as wisdom to others in wider society. Helen (aged 64) found sharing her views on positivity in life to be fulfilling and it helped her to reaffirm her meaning and extend her sense of being in old age:

*Helen: “So, my mantra is focus on strengths, which makes your weaknesses irrelevant … ‘we appreciate our strengths rather than constantly looking for problems?’ So that has become part of my thing here as well, when I’m talking to people. It’s very much reminding them of their life experiences and their strength and wisdom and things like that.”*

Despite this, we also noticed that many older people had not realised the power of life-story telling/sharing until the interview (much like Helen who felt the experience was eye-opening after talking about her fondness with her late parents), in which they were able to talk freely about their concerns and memories across their lifespan, helping them further appreciate their current experiences within the wider context of their biography:

*Interviewer: “Yes, it’s just such an extraordinary story.”*

*Helen: “Well, relating it to you makes me appreciate it more, because it’s something I just took for granted and never questioned. But the depth of it suddenly, talking to you, I thought oh wow, I must tell my brother and sister that.”*

By putting older people’s often under-recognised and undervalued stories into their broader biographical contexts, our analysis suggested that these older people faced a lack of accessible platforms to share their life stories and to reconstruct the continuity of their self-narratives. This may have been due to losses of long-lasting resources for emotional support alongside ageing. This situation was further compounded by the deprivation of a story-telling literacy in wider society where both older people and others had little knowledge about the benefits of sharing and listening to life stories. This issue was illuminated by Jennifer (aged 75), who lost cherished resources to face and deal with her long-lasting emotional pains. After seeing her adult children become independent and moving from a long-lived neighbourhood to a retirement village, she found:

Jennifer*:” This is what I miss a lot, a private space to talk … All my life I’ve suffered … and some things I do find very hard, like this illness now. With everything that’s gone wrong, I would have liked to talk to somebody, no advice, I want to let off steam. But it doesn’t happen…”*

It was only by adopting our biographical approach and enabling participants to tell their life stories so openly that the participants began to see the evident benefits/needs of storytelling. This methodological value lies in better capturing older people’s dynamic responses to varied emotional challenges and their attempts to regain the continuity of their self-narratives. The biographical lens could act as a ‘cathartic’ tool for the older people to understand their emotional needs in old age more accurately and acutely within their broader biographical context.

## Discussion

In this article, we adopted the Bath Loneliness Project as an example to examine how biographical storytelling can be a useful methodological tool to capture and centre older people’s emotional challenges within their life stories. Our analysis above has provided insight into the biographical nature of emotional challenges in old age. By adopting a broadly defined emotional experience of loneliness as a lens, we found that emotional challenges could arise from changes/losses that disrupt the continuity and coherence of older people’s ongoing construction of biography [3, 18]. As such, emotional challenges may be confronted as deeply painful experiences of standing alone in the present and being disconnected from their past and perceived future [25, 26]. Taking a biographical lens, we realised that these emotional challenges could run deeper, challenging the integrity of older people’s ongoing lives and the continuity of who they are. Despite the biographically embedded and often hard-to-address nature of these emotional challenges in old age, we further enriched this understanding by shedding light on these older people’s emotional needs, and more precisely how their emotional needs in old age may be shaped, undermined and restored, in alignment with their life histories and lived experiences. As such, our findings have illustrated both the risks to, and resilience of older people when facing emotional challenges [38]. More importantly, the biographical storytelling method has further highlighted the importance of understanding and supporting older people's emotional lives within their biography.

Our study has further highlighted the methodological value of biographical storytelling to collect and interpret older people’s emotional challenges. We believe this storytelling method is of particular relevance and importance to research older people’s experiences and needs as they tend to have accumulated more stories throughout their life histories that they may wish to share. By using our loneliness study as a showcase, we illustrated how a retrospective lens could be afforded in research design and interview collection to capture the biographical construction of older people’s emotional challenges. A biographical analysis approach was also exampled to show the methodological values of interpreting older people’s emotional needs within the wider context of their biography (and associated socio-historical backgrounds). Our methodological procedures and the findings, as highlighted in the case study, have reaffirmed the value of life-story interviews for understanding older people’s present experiences within their life histories [13, 17]. In addition, this article has further expanded the methodological scope of existing life-story interviewing methods by developing a more dedicated framework for researching (not seeking to change behaviour of) older people, who have rich life histories and may uniquely confront changes and challenges as they age [18].

Our exploration of biographical storytelling has also highlighted the continuity and fluidity of identity construction across the human lifespan, providing an insightful and integrative methodological tool to illustrate how identity construction continues into old age. Much emphasis has been laid thus far on identity development in earlier stages of life such as childhood, adolescence, or the broader stage of adulthood [39]. Conversely, old age is often seen as the last stage of life, and thus as reported by researchers and some older people themselves, ageing is closely connected a sense of tiredness and withdrawal from active human development of identity [40, 41]. By conducting a deep listening and reading exercise of the older people’s enriched life stories, we illuminated the older people’s dynamic needs and actions to (re)construct their identity alongside ageing (and even beyond physical death) and how these processes were shaped by their earlier life experiences. As such, we methodologically showcased the importance of connecting biographical gaps between earlier life stages and old age. That is, to provide a more longitudinal context to understand how the older people’s emotional lives have been continuously shaped by the ongoing construction of their meaning and identity throughout their lifespan, and more importantly how their emotional challenges in old age may arise from losing the consistency and integrity of their sense of self moving forward.

By depicting an insightful picture of our participants’ life stories, we further demonstrated the power of biographical storytelling to capture the nuanced experiences of emotional challenges in old age. Existing literature on qualitative methods in ageing studies has emphasised the importance of empowering older people to express their subtle and subjective emotions, feelings and needs using their own voices [8, 10]. This importance is particularly prevalent when exploring painful feelings. Our study furthered such methodological approaches by encouraging older people to reconstruct their life stories across their biography, including not only big stories (major or life-changing events such as, moving home, illness and bereavement) but also small stories as lived and enriched within everyday settings (e.g., daily interactions and conversations with others). Although small stories may be easily forgotten or overlooked, the focus on such subtle but meaningful experiences is of particular methodological significance to capture how small emotional turbulences may accumulate and unknowingly contribute to the ongoing construction of older people’s meaning and identity [11]. We afforded the older people a unique opportunity to freely and reflexively tell both their big and small life stories, with which they were able to provide more enriched materials to revisit, reveal and even re-appreciate their memories and feelings of pains, worries and fears in varied aspects. From a data analysis perspective, the sensitivity to small stories could also avoid over-simplification and over-categorisation of only connecting the nuanced and complex experiences of older people’s emotional challenges to grand narratives of their life, but not through the lens of their own lived experiences and smaller contributing stories that can accumulatively significantly shape constructions of self.

In addition to the above methodological benefits, our study has demonstrated the importance of developing appropriate tools and skills when implementing the biographical storytelling method with older people. First, a comprehensive and flexible interview schedule is key to allow for in-depth conversations about older people’s life stories while staying broadly on track about their emotional lives and challenges in old age. As shown in our study, the guide needs not to strictly follow a chronological trajectory but allow for the smooth progression of conversation to facilitate the development of a good rapport with the older people. Such rapport can enable researchers to gently walk older people through their lived lives to elicit recollections and reflections on their emotional ups and downs. The above caution about not too strictly prescribing an interview structure is also connected to the second requirement, that is to equip researchers (as interviewers) with transferable skills to learn how to be empathetic, supportive and focused throughout and across the often long-lasting interviews (e.g., over one hour, sometimes longer). Given the sensitive and even painful nature of emotional challenges in old age, training prior to initial interviews and frequent follow-up self/group reflection sessions may be of use to help researchers develop their own ways to deal with unexpected and challenging disruptions confronted during or from an interview (e.g., the participant suddenly bursting into tears or feeling distressed by certain conversations). Third, a biographical analytical lens is also needed for researchers (as interpreters) to examine older people’s emotional challenges within a wider context of their individual life histories and the associated social–historical circumstances. To gain such an integrative lens, researchers could benefit from developing in-depth theoretical understandings about the life course and the narrative construction of identity (see Fig. 1).

Finally, the insightful responses by these older people have also highlighted the merits of extrapolating the biographical storytelling approach outside of this research paradigm and in a more societal setting. Extant research [37] and our findings have both shown a lack of ‘safe and interested’ others and accessible platforms to facilitate life story telling between older people, their family, communities and wider society. Therefore, it would be of significant value to encourage older people and those in their support networks to further recognise the values of biographical storytelling and develop suitable knowledge about how to tell and/or listen to life stories [13]. We also believe that the practice of telling one’s life stories is not restricted solely to verbal forms (e.g., interviews or conversations) but has the potential to be developed through varied accessible and often creative forms depending older people’s preferences and capabilities. For example, the memory box approach as reported by Craig and Jacqueline in our study and other verbal and non-verbal mediums (e.g., music, painting, life-story writing, filming) may offer more diverse and participatory societal literacy to understand the circumstances, experiences and responses of older people’s emotional challenges and to further clarify their emotional needs as a biographical construct.

## Implications

By illuminating how we explored older people’s emotional challenges in the context of their life histories, our study has promoted further questions about the applicability of this biographical storytelling method. The value of this method elucidated throughout this paper suggests that this method may be more broadly valuable in the paradigm of ageing studies. Despite our primary finding that older people’s emotional barriers, pains and responses may be rooted in their experiences during earlier life, we are aware of a risk in future qualitative inquires of mis- and/or over-interpreting the retrospective elements of biographical narratives and the impacts this may have on older people’s nuanced emotional experiences. This could be manifested in the application of a psycho-analytical lens that too heavily stresses causalities between certain emotional challenges in old age and traumatic experiences in early life.

Instead of focusing on such a mechanical and prescriptive approach to linking specific and fragmented snapshots of experience between past and present, we feel it would be beneficial to embed more longitudinal elements into the biographical storytelling method to continuously follow up older people’s emotional experiences. For example, there is great potential to engage with cohort studies to use their longitudinal qualitative data often collected over years to explore the ongoing and dynamic construction of how cohort members deal with their emotional challenges as they grow older. Further, biographical storytelling interviews can be conducted with cohort members to collate stories (especially small stories) that have not been picked up by previous qualitative data collections. Likewise, where longitudinal data is not yet available for a research topic, the biographical storytelling methodology outlined above may begin to allow researchers to draw links between lived life and current experience in a way which is not afforded by non-biographical approaches.

## Limitations

A key limitation of this article is the example study lacks ethnic diversity within its interview sample, as only three non-white participants were recruited. As a result, our study had limited data to explore emotional challenges of older people from diverse backgrounds and how the unique socio-cultural environments they grew up and lived through may shape their emotional experiences and responses in facing changes/losses in old age. This racially homogeneous sample may have also prevented us from identifying any significant cultural differences between the participants in the UK and Australia and ultimately how biographical storytelling as a methodology should/can be addressed within different contexts. Therefore, future implementation and development of biographical storytelling in gerontology should endeavour to explore older people’s life stories with a more ethnically diverse sample and/or between more socio-culturally distinctive countries.

## Supplementary Information

**Additional file 1.**

## Data Availability

The datasets used in this article are available from the corresponding author upon reasonable request.
