# Supplementary material for: “They want you to know who they really are inside of the old visage”—biographical storytelling as a methodological tool to explore emotional challenges in old age
Source: BMC Geriatr. 2023 Jun 23;23:386. doi: 10.1186/s12877-023-04094-8 (PMC10290368; doi:10.1186/s12877-023-04094-8)
Supplement: Supplementary file 1 — Additional file 1. [file 12877_2023_4094_MOESM1_ESM.docx]

# **Appendix 1: interview structure and reflections on biographical storytelling of emotional challenges in old age**

**Introduction**

- This document outlines the interview structure and example questions of the Bath Loneliness Project. Instead of providing detailed questions, here we provide generalised understandings about how biographical interviews can be structured. As such, we aim to further inform readers to develop their own methodological approach to explore a diverse array of emotional challenges in old age. As can be seen below, the interview sections are biographically organised to encourage older people to reflect on both ‘big’ and ‘small’ stories across their lifespan.
- Section 1 and 2 focus on interviewees’ earlier life histories, while Section 3-5 are designed to explore their ageing lives and particularly their experiences of loneliness and how other emotional challenges may intersect with these to shape their everyday lives and responses to these challenges.
- Please note that we understand loneliness as more than simply the feelings associated with lacking meaningful relationships and interactions. Rather, we approach loneliness as a broadly defined condition that is deeply imbued with varied emotional challenges (e.g., experiencing loneliness may be a mix of feelings such as sadness, feeling forgotten and anxiety about health and changes). In so doing, we seek to use this broadly defined loneliness as a lens to showcase how emotional challenges in old age may be rooted in, and dealt with in ongoing narrative construction of biography.
- In addition to the interview structure, we have also included reflections from each of the sections to highlight techniques needed and potential challenges confronted during the interview.

| Interview structure | |
| --- | --- |
| Interview sections (including example guide questions) | **Methodological reflections** |
| Section 1: earlier adulthood  Work   - What is/was your work? How did you get into this line of employment? - Can you tell me about what your work is/was about? - Do/did you enjoy it? Why so?   Partner   - What is your marital status? - If partnered, how long have you been together? - If bereaved or divorced, when and how did it happen? - What is/was what role your partner/spouse is/was playing in your life? - Do you remember anything particularly special about your relationship?   Children   - Do you have any children? If so, how many? - How is/was your relationship with your children (each of them)? - Do you remember anything particularly special about the upbringing of your children? | - Section 1 aims to capture a broader picture of the older people’s earlier adult life histories and what they believed is important to them in their social, romantic and family lives. - To start with these broad questions about their earlier lives, we wanted to make it clear to the older people that we are interested in knowing all about their lived lives. - This impression could further nurture rapport with them and further enrich ongoing conversations with open and in-depth discussions about all aspects of their life stories. |
| Section 2: childhood and adolescence  Parents   - Are you parents still alive? How is/was your relationship with your parents? - Do you remember anything particularly special about your parents when you were growing up? - How did your relationship with your parents evolve as you grew older (into adolescence and further)?   Siblings   - Do/did you have any siblings? How is/was your relationship with your siblings? - Do you remember anything particularly special about your siblings when growing up with them? - How did your relationship with your siblings evolve as you grew older (into adolescence and further)?   Upbringing   - How would you describe your childhood and adolescence? - Did you have to face any significant challenges in your childhood/adolescence? Such as separate from parents/family, bullying, abuse or loss of close ones and relationships. - How did you deal with challenges in your childhood/adolescence, if any? | - Section 2 moved backwards to gain insights into the early development of the older people’s emotional attachment/detachment with others and how these experiences could continue to shape their interpersonal relationships and emotional lives. - Talking about childhood and adolescence could be potentially distressing or even traumatising. However, the rapport developed in section 1 helped us to gain a sense of trust from the older people so that they could feel safe and comfortable to share their experiences with us. |
| Section 3: emotional closeness of support figures in ageing lives  Close others in old age   - Do you have any family members (e.g., children, siblings, parents) or friends you see you regularly? If so, how would you spend time with them? - If you had a problem of some sort, would you turn to your partner/spouse? If so, how? If not, why? - Who else would you confide into when facing challenges? How? - What sort of things do you tell your close others? - Do you find it is hard to confide into him/her? Why so?   Recent life challenges and support networks   - Have you confronted any significant challenges recently? Such as illness, grief and other incidents. If so, could you tell me a bit more about what exactly happened. - If and how did you seek support from your networks to deal with these challenges? | - In Section 3, we were particularly interested in their emotional resources and capability by inquiring what support they had received or not received in facing recent difficulties (e.g., illness, bereavement and other incidents). - The life stories gained in section 1 & 2 provided a biographical grounding and formation to contextualise how their emotional support resources and resilience may be connected to their earlier life experiences and relationships. |
| Section 4: loneliness and other emotional challenges in old age  Loneliness   - Let’s talk a little more about the recent life challenges we talked about earlier. How did you feel when you go through these challenges? - In a sense, everyone is ageing. In your case, I wondered how you feel about you growing older. - Have you lost anyone/anything important to you alongside your ageing? If so, what? How this such impacts you and your emotional lives? - Reflecting on your loss, have you ever experienced a sense of disconnection? For example, lack of interactions with others, alienation from the life and the world you used to know.   Other/broader emotional challenges   - Do you have any particularly strong feelings of challenges in your current lives? If so, what? - When you look back at your life, is there anything that has changed over the years of your ageing? If so, what has it changed and how has it impacted you and the sense of who you are? - What do you miss the most from your earlier lives? If any, how do you cope with such significant voids in your ongoing lives. | - By following up the conversations on recent life challenges in section 3, we aimed to gently shift from an already well-discussed topic to ‘loneliness and other emotional challenges’, in order to reduce potential offence and harm to the older people. - This section was the key focus of the interviews, all the life stories so far could be used to help understand the older people’s current emotional pains within the context of their lived lives. - Key in the conversations was to explore how the varied loneliness and emotional challenges may be connected to losses of and voids in their life routines and taken-for-granted lives so far. |
| Section 5: living arrangements and future concerns/hopes  Living arrangements   - How long have you been living in your current residence? - What do you like/dislike by living here? - Are your living environments and social networks helpful for coping with the emotional challenges we previous discussed? - Have you gain anything positive from ageing in your living environments and social networks? If so, what is it?   Future concerns/hopes   - What can you envisage regarding your future ageing lives? - Is there anything you are concerned/worried about in relation to your future? If so, how would you address your concerns/worries? - Life is uncertain, but we can only live for now and stay positive about future. Do you have any hope or anything you look forward to in future? If so, what is it and why so? | - The final section focused on the older people’s responses to emotional challenges and how their actions and plans were informed by their long-lived ongoing lives. - These questions unfolded how the older people sought to retain/restore social and family connections and thus increase their support network. The focus on living arrangements could shed light on what is key to their ageing lives and how they plan to maintain it. - Section 5 afforded further understandings about their fundamental needs in old age, and how their needs may be interconnected to not only their past but also their future. - To minimise the impact of the potentially sensitive topics addressed during the interview, it is useful to finishing the interview with uplifting conversations (e.g., positive gains, hopes) |
